# Association between polymorphisms of *IL4, IL13, IL10, STAT6* and *IFNG* genes, cytokines and immunoglobulin E levels with high burden of *Schistosoma mansoni* in children from schistosomiasis endemic areas of Cameroon

**DOI:** 10.1016/j.meegid.2023.105416

**Published:** 2023-07

**Authors:** Estelle Mezajou Mewamba, Harry Noyes, Arnol Auvaker Zebaze Tiofack, Rolin Mitterran Ndefo Kamga, Cyrille Nguemnang Kamdem, Loic Edmond Tekeu Mengoue, Elvis Ofon, Romuald Isaka Kamwa Ngassam, Oscar Nyangiri, Bruno Bucheton, Flobert Njiokou, Macaire Hilaire Womeni, Enock Matovu, Annette MacLeod, Gustave Simo, for the TrypanoGEN+ research group of the H3Africa consortium

**Affiliations:** a Molecular Parasitology and Entomology Unit, Department of Biochemistry, Faculty of Science, University of Dschang, Dschang, Cameroon; b Centre for Genomic Research, University of Liverpool, Liverpool, United Kingdom; c Medical Laboratory Science, Faculty of Health Sciences, University of Buea, Buea, Cameroon; d Department of Biological sciences, Faculty of Science, University of Maroua, Maroua, Cameroon; e College of Veterinary Medicine, Animal Resources and Biosecurity, Makerere University, Kampala, Uganda; f Institut de Recherche pour le Développement (IRD), UMR INTERTRYP IRD-CIRAD, Campus international de Baillarguet, Montpellier, France; g Programme National de Lutte contre la Trypanosomose Humaine Africaine, Conakry, Guinea; h Parasitology and Ecology Laboratory, Department of Animal Biology and Physiology, Faculty of Science, University of Yaoundé I, Yaoundé, Cameroon; i Centre for Research in Infectious Diseases, Yaoundé, Cameroon; j Unité de Recherche de Biochimie, des plantes Médicinales, des Sciences alimentaires et Nutrition, University of Dschang, Dschang, Cameroon; k Institute of Biodiversity, Animal Health and Comparative Medicine, University of Glasgow, Garscube Estate, Glasgow, United Kingdom

**Keywords:** *Schistosoma mansoni*, Circulating cathodic antigen, Cytokines, IgE, Genetic polymorphism, Infection intensity

## Abstract

Eliminating schistosomiasis as a public health problem by 2030 requires a better understanding of the disease transmission, especially the asymmetric distribution of worm burden in individuals living and sharing the same environment. It is in this light that this study was designed to identify human genetic determinants associated with high burden of *S. mansoni* and also with the plasma concentrations of IgE and four cytokines in children from two schistosomiasis endemic areas of Cameroon. In school-aged children of schistosomiasis endemic areas of Makenene and Nom-Kandi of Cameroon, *S. mansoni* infections and their infection intensities were evaluated in urine and stool samples using respectively the Point-of-care Circulating Cathodic Antigen test (POC-CCA) and the Kato Katz (KK) test. Thereafter, blood samples were collected in children harbouring high burden of schistosome infections as well as in their parents and siblings. DNA extracts and plasma were obtained from blood. Polymorphisms at 14 loci of five genes were assessed using PCR-restriction fragment length polymorphism and amplification-refractory mutation system. The ELISA test enabled to determine the plasma concentrations of IgE, IL-13, IL-10, IL-4 and IFN-γ. The prevalence of *S. mansoni* infections was significantly higher (*P < 0.0001* for POC-CCA; *P = 0.001* for KK*)* in Makenene (48.6% for POC-CCA and 7.9% for KK) compared to Nom-Kandi (31% for POC-CCA and 4.3% for KK). The infection intensities were also higher (*P < 0.0001* for POC-CCA; *P = 0.001* for KK*)* in children from Makenene than those from Nom-Kandi. The allele C of SNP rs3024974 of *STAT6* was associated with an increased risk of bearing high burden of *S. mansoni* both in the additive (*p* = 0.009) and recessive model (*p* = 0.01) while the allele C of SNP rs1800871 of *IL10* was protective (*p* = 0.0009) against high burden of *S. mansoni*. The alleles A of SNP rs2069739 of *IL13* and G of SNP rs2243283 of *IL4* were associated with an increased risk of having low plasma concentrations of IL-13 (*P* *=* *0.04*) and IL-10 (*P* *=* *0.04*), respectively. This study showed that host genetic polymorphisms may influence the outcome (high or low worm burden) of *S. mansoni* infections and also the plasma concentrations of some cytokines.

## Highlights

• Prevalence and infection intensities of *S. mansoni* vary between sampling sites.
• Polymorphisms within *STAT6* and *IL10* genes were associated with high burden of *S. mansoni*.
• Low plasmatic concentration of IL13 and IL10 was associated with the polymorphim of *IL13* and *IL4* genes.
• Genetic polymorphisms of some cytokines influence *S. mansoni* infection outcomes and plasmatic concentrations of IL13 and IL10.
• Plasmatic concentration of IgE, IL13 and IFNG was correlated with the infection intensities of *S. mansoni*.

## 1 Introduction

Schistosomiasis is one of the most common neglected tropical disease (NTD) listed by the World Health Organization (WHO, 2020). It affects about 229 million people worldwide; whilst about 779 million are at risk of contracting the disease (WHO, 2018a; Quansah et al., 2021). >90% of schistosomiasis global burden occurs in Africa alone (Hotez and Kamath, 2009). This disease particularly affects children and ranks second only to malaria in terms of human suffering in the tropics and subtropics. Although the death estimates due to schistosomiasis needs to be re-assessed, its values have been reported to vary globally between 24,072 (WHO*,* 2018b) and 200,000 (WHO*,* 2002) per year. Moreover, an estimate of at least 236.6 million people required preventive treatment (WHO, 2023). Nevertheless, it is important to point out that large-scale preventive chemotherapy campaigns scale-up in most endemic areas over the past decades has probably reduced the morbidity and mortality linked to schistosome infections (WHO, 2023). Beyond its public health implications, schistosomiasis is regarded as one of the tropical parasitic diseases with greatest socioeconomic impact (Inobaya et al., 2014).

Preventive chemotherapy that relies on mass drug administration (MDA) of praziquantel (PZQ) to school-aged children (SAC) has considerably reduced the disease prevalence and currently, schistosomiasis has been included in WHO road map of neglected tropical diseases in which its elimination as a public health problem has been targeted for 2030 (WHO, 2020). Achieving this elimination requires to better understand the disease transmission. Although most inhabitants of schistosomiasis endemic regions share the same environment and are exposed to similar levels of schistosome infections, it has been shown that the intensity of infection varies widely between individuals and consequently, some individuals present high schistosome burdens while other harbor light burden. In addition, it has been demonstrated that >80% of eggs released in the environment come from around 25% of heavily infected population (Gurarie et al., 2015). These heavily infected people are therefore responsible of the majority of the eggs that are excreted in the environment and definitely have a great epidemiological impact on disease transmission.

Given the quantity of schistosome eggs released by heavily infected people in the environment and the impact of this on disease transmission, the elimination and the interruption of schistosome transmission will require to understand why some inhabitants of schistosomiasis endemic regions harbor high worm burdens than others. The factors underlying such differences as well as their implications for disease transmission are still not well understood. Although age, sex, contact frequency with water and host immunity can partially explain the difference in schistosome burdens (Abel and Dessein, 1997; Mutapi et al., 2008; De Moira et al., 2010), some host genetic determinants have been reported as potential factors that could play a role in the asymmetric distribution of parasite load and egg shedding between individuals (Bethony and Quinnell, 2008). Previous studies on Brazilian and Senegalese populations indicated that individuals with the heaviest burden of *S. mansoni* infections were grouped within families, rather than randomly distributed (Marquet et al., 1996; Müller-Myhsok et al., 1997). Results of these studies suggested that the susceptibility/resistance to high infections intensities may have some genetic components.

High infection intensity of *S. mansoni* should be probably controlled by a major locus SM1 (Abel et al., 1991) mapped to chromosome 5q31-q33 that contains three cytokine genes notably Interleukin 4 (*IL4*), Interleukin 13 (*IL13*) and Interleukin 5 (*IL5*) that are implicated in the Th2 immune response (Marquet et al., 1996; Marquet et al., 1999; Kouriba et al., 2005). These cytokines in turn influence Immunoglobulin E (IgE) levels and eosinophilia (Isnard and Chevillard, 2008; He et al., 2008) which have been associated with infection/re-infection or resistance to schistosome infections (Demeure et al., 1993; Caldas et al., 2000; Gatlin et al., 2009; Figueiredo et al., 2012; Ndombi et al., 2018). In addition to these three cytokines, polymorphisms in other genes encoding cytokines like Interleukin 10 (IL-10) and Interferon gamma (IFN-γ) involved in the activation of the Th1 immune response have also been associated with schistosome infections or re-infection (Grant et al., 2011; Gatlin et al., 2009). Indeed, several polymorphisms in the *IFNG*, *IL10*, *IL13*, *IL4*, *IL5*, *STAT6*, *CTLA4*, *FCN2*, *COLECC11*, *ABO* and *RNASE3* genes have been associated with schistosomiasis (Mewamba et al., 2021a). For instance, genetic variants in *IL13*, *IL4*, *IL10*, *IFNG* and *STAT6* genes were associated not only with infection intensities and re-infection of *S. mansoni* after treatment (Gatlin et al., 2009; Grant et al., 2011; Grant et al., 2012), but also with *S. haematobium* infections and its infection intensities (Kouriba et al., 2005; He et al., 2008; Isnard et al., 2011; Adedoja et al., 2018; Adedokun et al., 2018; Sarpong-Baidoo et al., 2021; Hanton et al., 2022). In addition to that, polymorphisms at some loci of *IL13, STAT6* and *IL10* genes have been associated not only with total IgE levels (Moller et al., 2007; Grant et al., 2011; Potaczek and Kabesch, 2012), but also with other helminthic infections such as *Ascaris* infections (Moller et al., 2007; Peisong et al., 2004; Moller et al., 2007; Figueiredo et al., 2013). Despite the fact that several studies reported some associations between host genetic polymorphisms with schistosome infections, contradictory results have been reported between countries (Kouriba et al., 2005; Isnard et al., 2011; Adedokun et al., 2018; Mewamba et al., 2021a). For instance, the SNP rs7719175 in *IL13* gene that has been associated with the susceptibility to *S. haemtobium* in Malian population revealed no association in Nigerian children (Adedokun et al., 2018; Isnard et al., 2011). In addition, the SNP rs2243250 in *IL4* gene associated with susceptibility to *S. haematobium* in Nigerian population showed no association in Malian populations (Adedokun et al., 2018; Kouriba et al., 2005). Most of these studies did not try to associate at the same time the polymorphism of genes involved in Th1 and Th2 responses during schistosome infections. As the outcome of these infections are multi factorials and could vary according to endemic regions, a better understanding of differences between children bearing high and low worm burden of *S. mansoni* requires in-depth investigations associating host genetic polymorphisms with infection intensities, cytokines and IgE levels in inhabitants from different epidemiological settings.

This study was designed to identify human genetic determinants associated not only with high burden of *S. mansoni,* but also with plasma levels of IgE and some cytokines in inhabitants of two schistosomiasis endemic areas of Cameroon.

## 2 Methodology

### 2.1 Ethics statement

The study was approved by the National Ethics Committee for Research on Human Health of the Ministry of Public Health of Cameroon with the reference number N°2019/02/1144/CE/CNERSH/SP. The review board of the Molecular Parasitology and Entomology Unit of the Department of Biochemistry of the Faculty of Science of the University of Dschang gave its approval. Field surveys were conducted in schools with the approval of the administrative authorities, school inspectors, directors and teachers. The objective of the study was explained to parents/guardians and children. On behalf of the family, a signed written consent was obtained from the head of the family and an assent was obtained from children of 10 to 14 years. Participants over 20 years gave their written consent.

Results of parasitological and immunological tests were communicated to parents or guardians and all children found with schistosome infections were treated with PZQ (40 mg/Kg body weight) following WHO recommendations (WHO, 2002).

### 2.2 Study area

**Fig. 1:**
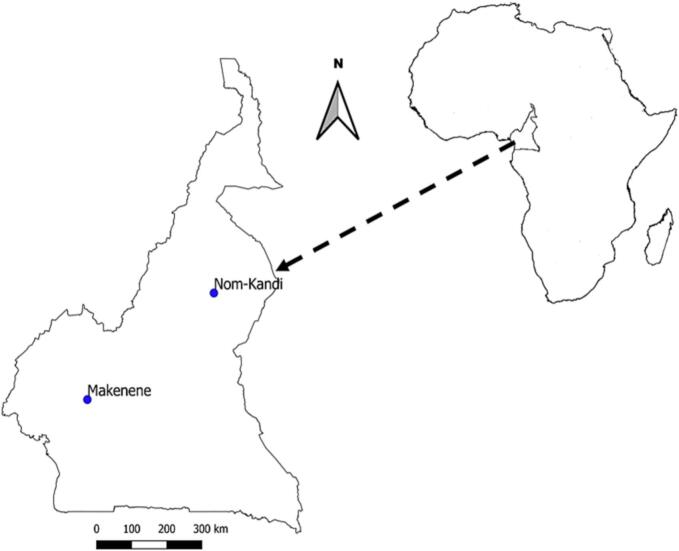
Map of Cameroon showing schistosomiasis foci where samples were collected.

This study was conducted in two schistosomiasis endemic areas of Cameroon: Makenene (4° 53′ 04″ N, 10° 47′ 40″ E) in the Centre region and Nom-Kandi (7° 32′ 00″N, 13°55′ 00″E) in the Adamawa region of Cameroon (Fig. 1).

- The locality of Makenene has an equatorial climate with 4 seasons: two dry seasons from November to March and from mid-May to mid-August and two rainy seasons from August to November and March to May. It has a dense hydrographic network containing several rivers (Mewamba et al., 2022). Inhabitants of this locality practice petty trading and farming, and the main crops are cassava, corn, groundnuts, yam, cocoa and palm nuts. *Schistosoma mansoni* infections have been reported in Makenene for >30 years (Ratard et al., 1990; Akufongwe et al., 1995; Tchuem Tchuenté et al., 2001).

- The locality of Nom-Kandi has a Sudano-Guinean climate with one dry season from November to March and one rainy season from April to October. It also has a dense hydrographic network containing several rivers. Its inhabitants practice agriculture, livestock breeding, fishing, beekeeping and trading. The main crops are cassava, corn, yams, potatoes, peanuts, beans and market garden products. Nom-Kandi belongs to the Adamawa region where schistosome infections have been reported for the past 30 years (Raccurt et al., 1987; Garba and Mbofung, 2010).

### 2.3 Sample collection

During this cross-sectional study conducted in schools of two endemic areas for schistosomiasis of Cameroon, each child from whom an assent and/or a signed informed consent form was obtained from their parents or guardians was invited to provide urine and stool samples in clean and well-labelled plastic containers that were given the sampling day. Approximately 50 mL of urine and 5 to 7 g of stool were collected from each child. These samples were immediately transferred to the local health centre where the Point-of-Care Circulating Cathodic Antigen (POC-CCA) and Kato-Katz (KK) tests were performed respectively on urine and stool samples to identify *S. mansoni* infections as described by Mewamba et al. (2022).

### 2.4 Diagnostic of *S. mansoni* infections

*Schistosoma mansoni* infections were detected and its intensity of infections determined using the Kato katz test in stool and the POC-CCA test on urine.

#### 2.4.1 Detection of *S. mansoni* eggs in stool samples

The protocol described by Katz et al. (1972) was used to search for *S. mansoni* eggs in stool samples. The intensity of *S. mansoni* infections was determined and expressed in eggs per gram of faeces (EPG) as described by WHO (2002).

#### 2.4.2 Detection of *S. mansoni* circulating cathodic antigen in urine samples

POC-CCA (Rapid Medical Diagnostics, Pretoria, South Africa, batch no 190411032) was used to diagnose *S. mansoni* infections by detecting Circulating Cathodic Antigens (CCA) as described by Mewamba et al. (2021b). The infection intensities or the amount of CCA in each urine sample was determined by reading each POC-CCA cassette with a lateral flow strip reader (ESEquant LR3 reader) as described by Mewamba et al. (2021b). This reader estimates the intensity of CCA on each POC-CCA cassette and expresses it in millivolts as described by the manufacturer (ESEquant LR3 User Manual, 2017). Each measurement was proportional to the intensity of the band reflecting the level of CCA on the strip or in the urine sample.

The top 20% of positive children of each community were considered as having a high level of CCA or high worm burden (Nyangiri et al., 2021). They were considered as cases during the genetic study while the remaining 80% of children positive to POC-CCA as well as those negative for this test were considered as controls during the assessment of plasma concentration of IGE and different cytokines. For the Transmission Disequilibrium Test (TDT), the main studied phenotype was children having high *S. mansoni* burden or cases. Children having this phenotype were those belonging to the top 20% of infected children. This top 20% was chosen because Woolhouse et al. (1995) have shown that the control programs targeting this percentage of people having high infection intensity distributions can be highly effective whilst those that fail to reach all these heavily infected people will have a much lower chance of success (Woolhouse et al., 1995).

### 2.5 Inclusion of nuclear families for genetic studies

After identifying children harbouring *S. mansoni* infections inferred from POC-CCA, the family of those bearing high *S. mansoni* burden (top 20% of children with positive POC-CCA or cases) were visited to obtain consent from parents and siblings of above 20 years old. An assent form was also obtained from children of 10 to 14 years. From infected children for whom parents and siblings accepted to participate to genetic studies by providing a signed inform consent and for whom assent forms were obtained, 5 mL of blood were collected into ethylene diamine tetra acetic acid (EDTA) coated tubes. From each of these tubes, 1 mL of blood was collected for DNA extraction and the remaining blood sample was used to prepare plasma samples.

### 2.6 Detection of *S. haematobium* infections

To exclude children harbouring co-infections of *S. haematobium* and *S. mansoni*, all urine samples that were positive with POC-CCA were tested to see if they have eggs of *S. haematobium*. This was performed using the urine filtration kit (Sterlitech Corporation, USA, Lot no: 070219) as described by the manufacturer. Each urine sample was vigorously shaken and thereafter, 10 mL were filtered through a 12 μm polycarbonate filter provided by the manufacturer. The filter was removed and placed on a microscope slide and a drop of Lugol's iodine solution was added to the slide before its microscopic examination. For each positive slide, the number of eggs was counted and expressed as number of eggs per 10 mL of urine.

### 2.7 DNA extraction

DNA was extracted from 1 mL of blood using “*Quick*-DNA Miniprep Plus Kit” (Cat.No:D4069, Lot.No:ZRC205022, Zymo Research, California, USA). Briefly, one mL of blood sample was put in a 2 mL tube. In this tube, 1 mL of nuclease free water was added and the tube was vigorously vortexed for 5 min. The mixture was centrifuged at 13000 rpm for 5 min. Thereafter, the supernatant was discarded and the pellet was re-suspended in 200 μL of phosphate buffer saline. The resulting suspension was used for DNA extraction as recommended by the manufacturer. DNA extract was stored at −20 °C until use.

### 2.8 Power calculation

It was performed as described by Purcell et al. (2003) using the genetic power calculator online software (https://zzz.bwh.harvard.edu/gpc/qtdt.html). The power of this study was estimated by considering a total Quantitative Trait Loci (QTL) ratio of 0.05, a frequency of the disease associated allele (ranging from 0.1 to 0.5), a frequency of the marker genotyped of 0.1, a linkage between disease SNP and the marker of 0.9, a case threshold of 1.25, a recombination fraction (0 for complete linkage to 0.5 for no linkage) of 0.01, the number of children with high worm burden and a type I error rate of 0.05.

### 2.9 Selection of candidate genes

For this study, fourteen SNPs located in five genes were identified and selected based on literature searches (Table 1). SNPs of *IL4, IL10, IL13, INFG* and *STAT6* genes were selected for their previous associations with *S. mansoni* or *S. haematobium* infections in terms of infection intensities, resistance to infections or re-infection (Table 1).

**Table 1:** Candidate genes and SNPs loci identified and selected for this study.

Genes	SNP	Localization	Characteristic	Reference
*IL10*	rs1800871	Chr1: 206773289	2 KB Upstream transcript Variant	(Grant et al., 2011)
*IL10*	rs1800872	Chr1:206773062	2 KB Upstream transcript Variant	(Grant et al., 2011)
*IL10*	rs1800896	Chr1: 206773552	2 KB Upstream transcript Variant	(Adedoja et al., 2018)
*IL13*	rs7719175	Chr5: 132650771	Intron Variant	(Isnard et al., 2011)
*IL13*	rs2069739	Chr5: 132656916	Intron Variant	(Isnard et al., 2011)
*IL13*	rs1800925	Chr5: 132657117	Intron Variant	(Kouriba et al., 2005; Gatlin et al., 2009; Grant et al., 2012; He et al., 2008; Isnard et al., 2011)
*IL13*	rs2069743	Chr5: 132657583	Intron Variant	(Kouriba et al., 2005)
*IL13*	rs1295687	Chr5: 132658770	Intron Variant	(Isnard et al., 2011)
*IL13*	rs20541	Chr5: 132660272	Coding sequence variant	(Grant et al., 2012)
*IL4*	rs2243250	Chr5: 132673462	2 KB Upstream Variant	(Gatlin et al., 2009; Adedokun et al., 2018)
*IL4*	rs2243268	Chr5: 132678271	Intron Variant	(Isnard et al., 2011)
*IL4*	rs2243283	Chr5: 132,680,901	Intron Variant	(Isnard et al., 2011)
*STAT6*	rs3024974	Chr12: 57098962	Intron Variant	(Adedokun et al., 2018)
*IFNG*	rs2430561	Chr12: 68158742	Intron Variant	(Gatlin et al., 2009)

Chr: Chromosome.

### 2.10 Genotyping of SNPs in *IL4*, *IL10*, *IL13* and *STAT6* genes

In this study, the polymorphisms at SNPs located in *IL4*, *IL10*, *IL13* and *STAT6* genes were investigated using PCR-Restriction Fragment Length Polymorphism in which DNA fragments of each of these genes were amplified before their digestion with specific restriction enzyme (Table 2).

**Table 2:** Expected sizes of PCR products and their digested fragments for each SNP.

				Genotypes			
Marker (Gene)	Amplification program	SA	RE	Heterozygote	Homozygote		References
					Wild type	Mutant type	
rs3024974T/C (*STAT6*)	94 °C, 5 min; 45 cycles; 94 °C, 30 s; 61 °C, 45 s; 68 °C, 45 s; and 68 °C, 5 min.	157	*Pst*I	157 bp/115 bp	115/32	157	(Adedokun et al., 2018)
rs2069743A/G (*IL13*)	94 °C, 5 min; 35 cycles; 94 °C, 30 s; 55 °C, 45 s; 68 °C, 45 s; and 68 °C, 5 min	313	*Drd*I	313/263/50	313	263/50	(Kouriba et al., 2005)
rs2243250C/T (*IL4*)		252	*BsmF*I	252/192/60	192/60	252	(Gyan et al., 2004)
rs1800925T/C (*IL13*)	94 °C, 5 min; 35 cycles; 94 °C, 30 s; 65 °C, 40 s; 68 °C, 50 s; and 68 °C, 5 min	247	*BstU*I	247/224/23	247	224/23	(Smolkova et al., 2017)
rs7719175T/G (*IL13*)	94 °C, 5 min; 35 cycles; 94 °C, 30 s; 55 °C, 40 s; 68 °C, 45 s; and 68 °C, 5 min	244	*Rsa*I	244/204/40	244	204/40	(Isnard et al., 2011)
rs20541A/G (*IL13*)	94 °C, 5 min; 35 cycles; 94 °C, 30 s; 55 °C, 30 s; 68 °C,45 s; and 68 °C, 5 min	236	*Nla*IV	210/178/32/26	178/32/26	210/36	(Kouriba et al., 2005; Graves et al., 2000)
rs2069739A/G (*IL13*)	94 °C, 5 min; 35 cycles; 94 °C, 30 s; 65 °C, 45 s; 68 °C, 45 s; and 68 °C, 5 min	365	*BseR*I	279/253/86/26	253/86/26	279/86	(Isnard et al., 2011)
rs1295687G/C (*IL13*)	94 °C, 5 min; 94 °C, 30 s; 60 °C, 30 s; 68 °C, 45 s; and 68 °C, 5 min; 35 cycles	495	*Dde*I	187/142/123/96/83/45/6	187/123/96/83/6	142/123/96/83/45/6	(Isnard et al., 2011)
rs1800872A/C (*IL10*)		482	*Rsa*I	482/258/224	482	258/224	(Song and Zhong, 2015)
rs1800896A/G (*IL10*)		139	*Mnl*I	139/106 /33	139	106/33	(Cai et al., 2015)
rs2243283C/G (*IL4*)		187	*HpyCh4*IV	187/117/70	117/70	187	(He et al., 2008)
rs2243268A/C (*IL4*)		232	*Nla*III	232/167/65	167/65	232	(He et al., 2008)
rs1800871T/C (*IL10*)	94 °C, 5 min; 35 cycles; 94 °C, 30 s; 60 °C, 30 s; 68 °C, 40 s; and 68 °C, 5 min;	593	*Msl* I	593/431/162	431/162	593	(Song and Zhong, 2015); An et al., 2015)

SA: Size of amplicons in base pair, RE: Restriction enzyme.

For each of these four genes, the amplification reactions were performed in a final volume of 25 μL containing 1× of PCR buffer, 1.5 mM MgCl_2_, 10 picomoles of each primer, 1.25 units of Taq DNA polymerase (New England Biolabs) and 5 μL of genomic DNA. The amplification programs are summarized in Table 2. The PCR products were resolved by electrophoresis on 2% agarose gel that was stained with ethidium bromide and then visualized under ultraviolet light.

Fifteen μL of PCR products were digested overnight at 37 °C (except *BstU*I at 60 °C) with restriction enzyme (all from New England Biolabs) (Table 2). The digested products were separated by electrophoresis on 3% agarose gel. This was performed at 100 V for 1 h 30 min. An internal control with known heterozygous genotype was included in each PCR-RFLP run.

### 2.11 Genotyping of SNP rs2430561 located in *IFNG* gene

Polymorphism in the *IFNG* gene was investigated as described by Albuquerque et al. (2009) using the amplification refractory mutation system polymerase chain reaction (ARMS-PCR). To amplify DNA fragment with allele A, PCR reaction was carried out in a total volume of 15 μL containing 5 μL of genomic DNA, 10 picomoles of generic primer (5′- TCAACAAAGCTGATACTCCA-3′), 10 picomoles of primer (5’-TTCTTACAACACAAAATCAAATCA-3′) specific to allele A, 200 mM of each dNTPs, 1× PCR buffer (Tris·Cl, KCl, (NH4)2SO4, 0.15 mM MgCl_2_) and 1.25 units of Taq DNA polymerase (New England Biolabs). In each PCR tube, DNA fragment of human growth hormone were amplified as internal control using two specific primers (forward primer: 5’-GCCTTCCCAACCATTCCCTTA-3′ and reverse primer: 5’-TCACGGATTTCTGTT GTGTTTC-3′). The amplification of DNA fragment having the allele T was performed as for the allele A. However, instead of using primer for allele A, another primer (5’-TTCTTACAACACAAAATCAAATCT-3′) was used.

The amplification program for the ARMS-PCR contained an initial denaturation step at 95 ^°C^ for 5 min followed by 10 cycles comprising a denaturation step at 95 °C for 30 s, an annealing step at 62 °C for 50 s and an elongation step at 68 °C for 40 s. This was followed by 20 amplification cycles comprising a denaturation step at 95 °C for 20 s, an annealing step at 56 °C for 50 s and an elongation step at 68 °C for 50 s and then, a final elongation at 68 °C for 5 min.

The amplified products were resolved by electrophoresis at 100 V for 45 min on 2% agarose gel containing ethidium bromide. The gels were visualized under ultraviolet light and then photographed using a gel documentation system (UVIsave HD5, Unitec Cambridge). PCR products were 261 bp for either allele A or T at SNP rs2430561 of *IFNG* gene and 429 bp for human growth hormone amplified as internal control. For heterozygote, amplifications were obtained for alleles A and T while for homozygote wild type and mutant, amplifications were obtained respectively for allele T and allele A.

### 2.12 Determination of cytokines and total IgE concentration in the plasma

For this determination, two phenotypes were used: the first one was made up of the top 20% of children having high infection intensity or cases; the second phenotype or control was formed by all uninfected children and those belonging to the remaining 80% of infected children. In these two groups of participants, plasma concentrations of human IL-13, IL-10, IL4, IFN-γ and IgE were measured as described by the manufacturer using human IL-13, IL-10, IL-4, IFN-γ and IgE Elisa kit (all from STEMCELL Technologies, Canada). Briefly, 96-well plates provided by the manufacturer were washed 5 times by introducing, in each well, 300 μL of washing buffer diluted 20 folds in deionized water. Thereafter, a serial dilution of each reconstituted standard was done as described by the manufacturer. One hundred microliters of diluted standards of IgE and each cytokine were added in the wells of the first raw of the plate. For IL-13, IL-10, IL-4 and IFN-γ plates, 100 μL of sample diluted 2 folds were added per well while for IgE plate, 100 μL of each sample diluted 100 fold were introduced into each well. For each plate, 100 μL of ELISA buffer were added in two wells that were used as negative controls. Each Plate was covered with adhesive plate covers and then, incubated at room temperature for 2 h. Thereafter, the plates were washed 5 times with washing buffer and then, 100 μL of biotinylated antibody diluted 1000 folds for IL-13, IL-10, IL-4, IFN-γ and 3333 folds for IgE were added in each well. The plates were incubated at room temperature for one hour, then wash before adding 100 μL of avidin-horseradish peroxidase solution diluted 1000 folds. The plates were incubated at room temperature for 1 h and then, washed before adding 100 μL of substrate (3, 3′, 5, 5’-Tetramethylbenzidine; TMB) in each well. The plates were incubated in the dark for 15 min. The reactions were stopped by adding, in each well, 100 μL of stop solution provided by the manufacturer.

The absorbance was read at 450 nm in each well using an ELISA plate reader (Diagnostic Automation,Inc., DAR800, USA). Each absorbance or optical density was converted into antibody and cytokine concentrations using Graphpad Prism version 9.3.1 software (Dotmatics, San Diego, California USA, www.graphpad.com).

### 2.13 Statistical analysis

#### 2.13.1 IgE and cytokine concentrations, *S. mansoni* prevalence and its infection intensities

The chi square test was used to compare the prevalence of *S. mansoni* between sample sites, age and sexes. The Kolmogorov–Smirnov test was used to check if the data of the infection intensities of *S. mansoni* were normally distributed. The Mann-Whitney *U* test was subsequently used to compare not only the means of infection intensities (expressed in EPG or mV) between sampling sites as well as sexes, but also the means of log transformed plasma concentrations of cytokines and CCA amount between cases and controls. The Kruskall Wallis H test enabled to compare the means of infection intensities (expressed in millivolts) according to age. The test was considered significant when the *p*-value was below 0.05.

#### 2.13.2 Family based association studies analysis

The transmission disequilibrium test (TDT) and the quantitative transmission disequilibrium test (QTDT) of family based association tests were used to assess the association between genetic polymorphism and infection intensities inferred from the POC-CCA test.

##### 2.13.2.1 Association study using transmission disequilibrium test (TDT)

For this association study, only children with phenotype formed by high worm burden or belonging to the top 20% of positive POC-CCA were used while other children were excluded during the analysis. The TDT was used to see if there is a linkage between a genetic marker and a specific trait (Spielman et al., 1993; Spielman and Ewens, 1998). The association between genetic polymorphism and high *S. mansoni* burden was assessed using TDT analysis implemented in the Family-Based Association Test Package (FBAT, version 3.5.0) (Laird et al., 2000). Any test with a p-value of <0.05 was considered significant.

##### 2.13.2.2 Association study using quantitative transmission disequilibrium test (QTDT)

For the QTDT, three phenotypes were used: infection intensities (amount of CCA inferred from POC-CCA), the concentrations of cytokines (IL-4, IL-10, IL-13, and IFN-γ) and IgE. In addition to these phenotypes, some confounding factors such as age, gender and study site were considered during the QTDT. The effect of each confounding factors (age, sex and sampling site) on different phenotypes was assessed using the linear regression model as previously described (Grant et al., 2012; Lunetta et al., 2000). This assessment was performed using R-software version R3.6.1. Associations between individual polymorphisms and each of the three phenotypes were assessed using the Family-Based Association Test Package (FBAT; version 3.5.0). The test was considered significant when the p-value was below 0.05.

For all the association studies, no correction for multiple testing or Bonferroni correction was performed in this study.

## 3 Results

### 3.1 Epidemiological data

A total of 3743 (3012 from Makenene and 731 from Nom-Kandi) urine and 3220 (2532 from Makenene and 688 from Nom-Kandi) stool samples were collected from school-aged children in two schistosomiasis endemic regions of Cameroon. The overall prevalence of *S. mansoni* infections were 45.2% inferred from POC-CCA test and 7.1% from KK test (Table 3). This prevalence was significantly higher (*χ*^*2*^ *= 73, P < 0.0001* for POC-CCA; *χ*^*2*^ *= 9.68, P = 0.001* for KK test*)* in Makenene (48.6% for POC-CCA and 7.9% for KK) compared to Nom-Kandi (31% for POC-CCA and 4.3% for KK). The infection intensities were significantly higher (*U = 687,849.5*, *P < 0.0001* for POC-CCA; *U = 838,494*, *P = 0.001* for KK test*)* in children of Makenene compared to those of Nom-Kandi (Table 3).

**Table 3:** Prevalence and infection intensities of *S. mansoni* according to sampling sites.

			Prevalence		Infection intensities	
Study site	NUA	NSA	POC-CCA^+^ (%)	KK^+^ (%)	Mean CCA (mV)	Mean EPG
Makenene	3012	2532	1466 (48.6)	200 (7.9)	94.12	37
Nom-Kandi	731	688	227 (31)	30 (4.3)	33.29	2
Total	3743	3220	1693 (45.2)	230 (7.1)	82.24	30
P			P < 0.0001	0.001	P < 0.0001	0.001
χ^2^			73	9.68	–	–
U			–	–	687,849.5	838,494

NUA: Number of urine samples analyzed; POC-CCA^+^: Number of children positive for POC-CCA test; KK^+^: Number of children positive for the Kato Katz test; EPG: eggs per gram of stool; Mean CCA: Mean of circulating cathodic antigen in overall school children screened; Mean EPG: Mean of eggs per gram of stool; NSA: Number of stool samples analyzed. P: *P* value, χ^2^ and U correspond to values of chi-squared and Mann Whitney *U* test.

**Fig. 2:**
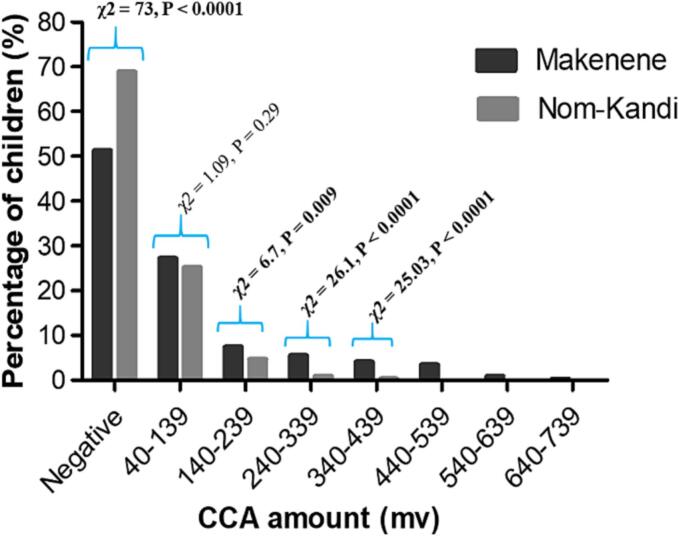
Distribution of POC-CCA results according to study site.

The proportion of children with negative POC-CCA was significantly higher (*χ2 = 73, P < 0.0001*) in Nom-Kandi (68.9%) compared to Makenene (53.1%) (Fig. 2). Regarding children having CCA in their urine, their proportion vary significantly between the study sites, except for those having 40 to 139 mv (*χ2 = 1.09, P = 0.29*) (Fig. 2). Children with CCA amount above 440 mV were only found in Makenene (Fig. 2).

CCA: Circulating cathodic antigen, mV: millivolts.

The highest prevalence of *S. mansoni* infections of 52.6% was found in children of 12 years and the lowest prevalence of 35% in those of 15 years (Table 4). Comparing these prevalence, significant difference (*χ2 = 19.26*, *P = 0.03*) was recorded between age groups. For the mean of infection intensities, significant difference (*H = 66.19*, *P < 0.0001*) was also recorded between age groups; children of 14 years had the highest value of 271.7 ± 196.9 mV while those of 6 years had the lowest value of 127 ± 115.5 mV (Table 4).

**Table 4:** Prevalence and infection intensities of *S. mansoni* according to age and sex.

Category	NUA	POC-CCA+ (%)	Mean CCA ± SD (mV)
Age			
5	235	103 (43.8)	127.1 ± 114.8
6	678	289 (42.6)	127 ± 115.5
7	511	213 (41.7)	160.3 ± 139.2
8	518	224 (43.2)	154.1 ± 124.1
9	467	216 (46.3)	185.2 ± 151.3
10	525	247 (47)	200.5 ± 165.9
11	329	170 (51.7)	195.8 ± 153.3
12	251	132 (52.6)	205.3 ± 158.1
13	128	59 (46.1)	190.3 ± 159.2
14	61	26 (42.6)	271.7 ± 196.9
15	40	14 (35)	163.5 ± 132.5
Total	3743	1693 (45.2)	170.7 ± 145.9
		**χ*2 =* 19.26; *P =* 0.03**	***H =* 66.19; *P <* 0.0001**
Sex			
Male	1946	922 (47.4)	177.3 ± 149.7
Female	1797	771 (42.9)	162.7 ± 140.9
Total	3743	1693 (45.2)	170.7 ± 145.9
		**χ*2 =* 7.37, P = 0.006**	**U = 338,043.5, P = 0.08**

SD: standard deviation; Mean CCA: Mean of circulating cathodic antigen; NUA: Number of urine samples analyzed; POC-CCA^+^: Number of children positive for POC-CCA test, **χ*2*** = chi-square test, H: Kruskal Wallis H test, U: Mann Whitney *U* test.

The prevalence of *S. mansoni* infections was significantly (χ2 = *7.37*, *P = 0.006*) higher in boys (47.4%) than in girls (42.9%). For the infection intensities, no significant difference (*U = 338,043.5*, *P = 0.08*) was observed between boys (177.3 ± 149.7 mV) and girls (162.7 ± 140.9 mv) (Table 4).

Two children from Makenene were infected by *S. haematobium* and none from Nom-Kandi.

### 3.2 Characteristics of participants used for the genetic analysis

From the 1693 children who were positive to POC-CCA test, 340 were considered as cases. From these 340 cases, the consent to participate to genetic studies was obtained from 115 parents or guardians of nuclear families. From these 115 families, 462 individuals comprising 157 cases, 85 controls, 170 parents, 50 unphenotyped siblings (used to infer genotypes of missing parents) were retained for genetic studies. Out of these 115 families, a total of 157 trios (made up of father, mother and case or infected child carrying high worm burden) were formed for FBAT. Among the 462 individuals retained for genetic studies, only 242 of them having POC-CCA results (positive or negative) were used for QTDT.

Comparing the infection intensities (means of CCA amount) in children involved in genetic association studies, significant difference was recorded between cases and controls (*U = 514; P* *< 0.0001*) (Table 5). Between these two groups of children, significant differences were also recorded for the concentration of IgE (*U* *=* *1119, P = 0.003*) as well as that of IL-13 (*U* *=* *1448, P = 0.003*) (Table 5). However, no significant difference was recorded for the concentration of IL-10 (*U = 2365.5, P = 0.46*), IL-4 (*U = 2504.5, P = 0.87*) and IFN-γ (*U = 2126.5, P = 0.09*) (Table 5).

**Table 5:** Means of CCA, cytokines and IgE concentrations according to POC-CCA results.

Phenotype	Cases (N)	Controls (N)	P or U
Mean CCA amount ± SD	377.9 ± 127.8	90.9 ± 92.7	U = 514, P < 0.0001
Mean concentration of IgE and cytokines in logarithm ± SD			
IgE	2.82 ± 0.49 (48)	2.47 ± 0.67 (69)	U = 1119, P = 0.003
IL-13	2.10 ± 0.51(58)	1.8 ± 0.58 (71)	U = 1448, P = 0.003
IL-10	2.17 ± 0.39 (76)	2.20 ± 0.54 (67)	U = 2365.5, P = 0.46
IL-4	1.14 ± 0.36 (76)	1.10 ± 0.31 (67)	U = 2504.5, P = 0.87
IFN-γ	1.11 ± 0.33 (76)	1.03 ± 0.34(67)	U = 2126.5, P = 0.09

Cases: top 20% of children with positive POC-CCA tests, Controls: the remaining 80% of children with positive POC-CCA and those with negative POC-CCA test; N: number children; SD: standard deviation; P: *P*-value; U: Mann Whitney U test.

### 3.3 Power of the study

With a total QTL ratio of 0.05, a linkage disequilibrium value of 0.9, the disease allelic frequencies ranging from 0.1 to 0.5, a case threshold of 1.25, a recombination fraction of 0.01, and 157 children bearing high worm, the power of our study ranged from 85.6% to 89% (Supplementary Table S1).

### 3.4 Results of transmission disequilibrium test

The study population was in Hardy-Weinberg equilibrium at all SNPs (Supplementary Table S2). In the additive and recessive models, the allele C of SNP rs3024974 located on *STAT6* gene was associated with an increased risk of bearing high worm burden or high infection intensities (*Z* = 2.61, *P* = 0.009 for additive model; *Z* = 2.55, *P* = 0.01 for the recessive model) (Table 6). The allele C of SNP rs1800871 of *IL-10* gene was associated with a decrease risk of bearing high infection intensities in a recessive model (*Z* = −3.3, *P* = 0.0009). However, no association (*Z* = 1.21, *P* = 0.22) was observed for this allele in the additive model (Table 6). When association studies were performed according to sampling size, the allele C of SNP rs3024974 of *STAT6* (Z = 2.80, *P* = 0.004) was significantly more associated in population from Makenene in the additive and recessive models (Supplementary Table S3). The allele C of SNPs rs1800871 of *IL10* remained significantly associated (Z = −2.80, *P* = 0.005) with a decrease risk of having high worm burden in the recessive model for population of Makenene (Supplementary Table S3). However, no SNP of *STAT6* and *IL10* genes was associated with a risk of having high worm burden at Nom-Kandi (Supplementary Table S3).

**Table 6:** Results of association between polymorphisms at *IL4, IL10, IL13, IFNG* and *STAT6* genes with infection intensities of *S. mansoni.*

						Additive model			Recessive model		
Gene	Marker	Risk Allele	Allele frequency ^a^	A1	MAF	Informative families ^b^	Z Score ^c^	P	Informative families ^b^	Z Score ^c^	P
***STAT6***	**rs3024974**	C**	**0.89**	**T**	**0.11**	**37**	**2.61**	**0.009**	**35**	**2.55**	**0.01**
*IL13*	rs2069743	A	0.77	G	0.23	55	1.67	0.09	49	0.99	0.32
*IL4*	rs2243250	T	0.74	C	0.26	57	0.03	0.97	47	−1.10	0.27
*IFNG*	rs2430561	A	0.81	T	0.19	48	0.98	0.33	44	0.63	0.53
*IL13*	rs1800925	C	0.57	T	0.43	75	−0.14	0.89	55	−0.99	0.32
*IL13*	rs7719175	T	0.82	G	0.18	47	0.39	0.68	43	0.59	0.55
*IL13*	rs20541	G	0.88	A	0.12	38	−0.42	0.67	37	−0.44	0.66
*IL13*	rs2069739	A	0.60	G	0.40	76	−0.97	0.33	55	−1.62	0.10
*IL13*	rs1295687	G	0.66	C	0.33	65	0.60	0.54	52	0.40	0.68
***IL10***	**rs1800871**	**T**	**0.55**	C*	**0.45**	67	1.21	0.22	**36**	**−3.3**	**0.0009**
*IL10*	rs1800872	C	0.54	A	0.46	74	−1.28	0.20	54	−1.51	0.13
*IL10*	rs1800896	A	0.66	G	0.34	63	1.15	0.25	49	0.87	0.38
*IL4*	rs2243268	A	0.66	C	0.34	74	−0.64	0.52	64	−1.16	0.24
*IL4*	rs2243283	G	0.76	C	0.24	61	−1.13	0.26	54	−1.37	0.06

a: Calculated from the genotype of parents; b: Families with at least one heterozygous parent; c: Z = (S - E (S))/Var(S), where S is the test statistic (i.e. observed), E(S) is the expected value according to the null hypothesis (H0), and Var(S) is the variance of the statistic test according to H0; A1: Minor allele; MAF: Minor allele frequency, **: allele associated with an increase of bearing high worm burden, *: allele associated with a decrease of bearing high worm burden.

In the dominant model, no SNP was associated with high *S. mansoni* burden.

### 3.5 Results of the quantitative Transmission disequilibrium test

Results of association between the fourteen genetic markers and the CCA amount are displayed in Table 7. No association was found between these genetic markers and CCA amount.

**Table 7:** Results of quantitative transmission disequilibrium test associating polymorphism at 14 SNPs and CCA amount, IgE, IL-13, IL-4, IL-10 and IFN-γ plasma levels.

			CCA amount (mV)			IgE^a^			IL-10^a^			IL-13^a^			IL-4^a^			IFN-γ^a^		
Gene	Marker	Allele	P	Dir	NO	P	Dir	NO	P	Dir	NO	P	Dir	NO	P	Dir	NO	P	Dir	NO
*STAT6*	rs3024974	C	0.88	Neg	37	0.59	Neg	26	0.68	Neg	29	0.14	Pos	28	0.13	Neg	28	0.80	Neg	29
*IL13*	rs2069743	A	0.09	Pos	56	0.35	Neg	33	0.47	Neg	34	0.65	Pos	35	0.34	Neg	34	0.90	Pos	34
*IL4*	rs2243250	T	0.46	Neg	58	0.99	Neg	34	0.60	Pos	37	0.67	Neg	37	0.83	Neg	37	0.45	Pos	37
*IFNG*	rs2430561	A	0.17	Neg	50	0.66	Neg	32	0.59	Pos	37	0.98	Neg	35	0.71	Neg	37	0.12	Neg	37
*IL13*	rs1800925	C	0.64	Neg	77	0.10	Neg	43	0.74	Neg	46	0.64	Pos	47	0.83	Neg	46	0.78	Neg	46
*IL13*	rs7719175	T	0.10	Pos	48	0.53	Neg	28	0.94	Pos	32	0.85	Pos	30	0.67	Neg	32	0.67	Pos	32
*IL13*	rs20541	G	0.12	Neg	38	0.81	Pos	27	0.19	Neg	26	0.61	Neg	29	0.23	Neg	26	0.68	Neg	26
***IL13***	**rs2069739**	**A**	0.81	Neg	77	0.44	Neg	48	0.84	Neg	47	**0.04**^**b**^	Neg	51	0.25	Neg	46	0.96	Neg	47
*IL13*	rs1295687	G	0.61	Pos	67	0.40	Neg	42	0.36	Neg	44	0.05	Pos	45	0.89	Neg	43	0.89	Pos	44
*IL10*	rs1800871	T	0.69	Pos	69	0.69	Neg	42	0.35	Neg	48	0.96	Pos	46	0.39	Neg	48	0.40	Pos	48
*IL10*	rs1800872	C	0.05	Neg	77	0.28	Neg	42	0.17	Pos	44	0.40	Neg	46	0.65	Pos	44	0.33	Neg	44
*IL10*	rs1800896	A	0.12	Pos	65	0.18	Pos	36	0.18	Neg	39	0.48	Pos	39	0.33	Neg	38	0.93	Pos	39
*IL4*	rs2243268	A	0.99	Pos	78	0.33	Pos	44	0.21	Neg	45	0.57	Pos	47	0.78	Pos	44	0.50	Pos	45
***IL4***	**rs2243283**	**G**	0.37	Neg	65	0.35	Pos	41	**0.04**^**b**^	Neg	42	0.57	Neg	43	0.72	Pos	42	0.58	Neg	42

Dir: direction of association; pos: positive; neg: negative; NO: number of informative offspring; IgE: IgE; IL-10: interleukin 10 levels; IL-13: interleukin 13 levels; IL-4: interleukin 4 levels; IFN-γ: interferon gamma levels; ^a^: log (10)-transformed values adjusted for age, sex, and sampling site; ^b^: *P*-values in bold are statistically significant (*P* < 0.05).

The alleles A of SNP rs2069739 located in *IL13* gene and G of SNP rs2243283 of *IL4* gene had Z values respectively of −1.994 and − 1.998 with *P* values of 0.04 for each of them (Table 7). These alleles are associated with an increased risk of having low levels of IL-13 and IL-10. For IgE, IL-4 and IFN-γ, no association was recorded between polymorphism of these genes and their concentrations in the blood.

### 3.6 Correlation between the concentrations of cytokines and IgE with infection intensities

**Fig. 3:**
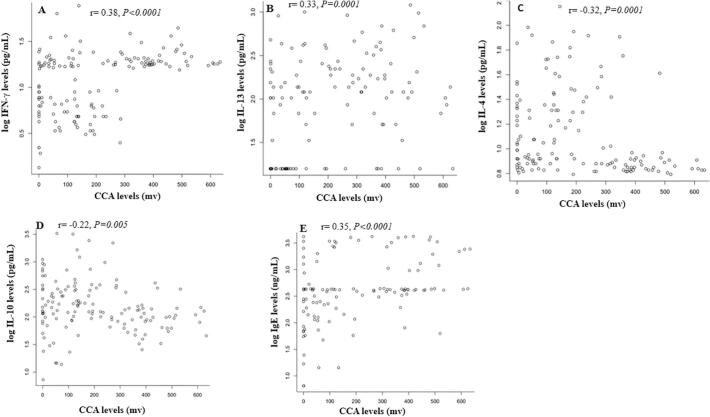
Correlations between (A) IFN-γ, (B) IL-13, (C) IL-4, (D) IL-10 and (E) IgE with CCA levels. ‘r’ denotes the Spearman's rank correlation coefficient (Spearman's rho).

Positive and significant correlations were obtained between the amount of CCA in children and the concentration of IFN-γ (*Spearman rho = 0.38, P < 0.0001*) as well as that of IL-13 (*Spearman rho = 0.33, P = 0.0001*). However, negative and significant correlations were obtained between the amount of CCA and the level of IL-4 (*Spearman rho = −0.32, P = 0.0001*) as well as that of IL-10 (*Spearman rho = −0.22, P = 0.005*). The concentration of IgE was positively correlated (*Spearman rho* *=* *0.35, P* *<* *0.0001*) with CCA levels (Fig. 3).

## 4 Discussion

This study was carried out with the primary aim of identifying human genetic determinants associated with high burden of *S. mansoni* and also with the plasma concentrations of IgE and some cytokines in school-aged children from two schistosomiasis endemic areas of Cameroon. The identification of *S. mansoni* infections by KK or POC-CCA confirmed that Makenene and Nom-Kandi remain endemic for schistosomiasis despite more than two decades of mass administration of PZQ to school-aged children. The schistosomiasis prevalence of 7.9% and 4.3% inferred from KK in school-aged children respectively from Makenene and Nom-Kandi are lower than 23.6% and 49.0% reported 27 and 32 years ago in Makenene. These prevalence are also lower than 54.4%, 24.1% and 27.8% reported in order endemic regions of Cameroon (Moyou-Somo et al., 2003; Njiokou et al., 2004; Raccurt et al., 1987). Even with the POC-CCA, the prevalence of 48.6% and 31.0% recorded in school-aged children respectively of Makenene and Nom-Kandi were lower than 69.8% obtained ten years ago in schistosomiasis endemic regions of Cameroon (Tchuem Tchuenté et al., 2012). The overall reduction observed in the schistosomiasis prevalence may result from the mass drug administration of PZQ to school-aged children of most schistosomiasis endemic regions of Cameroon since 2007 (Tchuem Tchuenté et al., 2013).

The prevalence of *S. mansoni* infections inferred from KK of 7.9% and 4.3% in school-aged children respectively from Makenene and Nom-Kandi are too low compared to 48.6% and 31% inferred from POC-CCA. The differences observed could be explained by: i) the ability of the POC-CCA to identify individuals carrying young worm's not yet shedding eggs (Shane et al., 2011; Colley et al., 2013; Coulibaly et al., 2013; Casacuberta et al., 2016); ii) the daily fluctuations in the number of eggs excreted; iii) the heterogeneous distribution of eggs in stool samples (Kongs et al., 2001; Berhe et al., 2004).

Results of association studies obtained with the recessive and additive models revealed that the C allele of the rs3024974 SNP in *STAT6* was significantly associated with an increased risk of bearing high burden of *S. mansoni* in Cameroon while the T allele protects against the risk of having high infection intensities. These results are consistent with those of Adedokun et al. (2018) who showed that the T allele of the same SNP was more frequent in uninfected children than infected ones. These results indicate that the allele T potentially protects against infection as well as high worm burden. Other polymorphisms located in the promoter region or other regions of *STAT6* gene have been associated not only with schistosomiasis in Mali (He et al., 2008), but also with *Ascaris lumbricoides* infections (Moller et al., 2007; Peisong et al., 2004). These results highlight the importance of polymorphisms of *STAT6* gene during helminth infections. As *STAT6* gene is involved in the regulation of Th2 immune response, results highlighting association between genetic polymorphism within this gene with the outcome of helminth infections suggest that these polymorphisms may probably play an important role in the regulation (Th2) of helminth infections.

The strong association recorded between gene polymorphism and high worm burden in children from Makenene compared to those from Nom-Kandi could be related to the genetic variations between the study populations. Children from Nom-Kandi seems to control *S. mansoni* infections more than those from Makenene. This capacity of controlling *S. mansoni* infections has been highlighted by the fact that the highest value (411.38 mV) of CCA in children from Nom-Kandi was about two times lower than the value (720.27 mV) obtained in children from Makenene. This hypothesis is strengthened by results of QTDT reporting no association between genetic polymorphisms and confounding factors such as sampling site, age and sex. The differences recorded in the results of association studies between Makenene and Nom-Kandi could be more likely related to the genetic polymorphism between inhabitants of the two schistosomiasis endemic areas. However, other factors interfering with schistosome transmission such as the density of infected snails, the contact frequency between children and infected snails, the frequency of Praziquantel distribution and the children behavior could not be ruled out. These factors may have some impacts in the transmission of schistosomiasis and hence, in the variations observed in the infection intensities of *S. mansoni* between the two sampling sites.

Results of the recessive model showing that the allele C of SNP rs1800871 of *IL10* was associated with protection against the risk of having high burden of *S. mansoni* are in accordance with those of Marume et al. (2021) who reported an association between polymorphisms at this SNP with susceptibility to *S. haematobium* infections. This C allele of SNP rs1800871 of *IL10* might control *S. mansoni* infection intensities. However, with the additive model, no association was obtained for all SNPs (rs1800871 or rs1800872 or rs1800896) genotyped on *IL10* gene*.* These results are in line with those of previous studies (Grant et al., 2011; Adedoja et al., 2018; Adedokun et al., 2018). The discrepancies observed between results of association studies involving SNPs located in *IL10* gene could be linked to the model of inheritance. In the additive model, both alleles contribute to the phenotype while in the recessive one, only one allele contributes to the phenotype studied.

Our results showing no association between infection intensities and polymorphisms at SNPs located on *IL4*, *IL13* and *IFNG* contrast with those of previous authors who reported some association between polymorphism of these genes in Brazilian (Grant et al., 2012), Malian (Kouriba et al., 2005; He et al., 2008; Isnard et al., 2011), Nigerian (Adedokun et al., 2018), Ghanaian (Sarpong-Baidoo et al., 2021) and Zimbabwean populations (Hanton et al., 2022). The discrepancies between results of the present study and those of previous ones could be explained by; i) the differences in allele frequencies within and between populations (Gomez et al., 2014; Walakira et al., 2017); ii) the genetic diversity within and between populations from different countries (Gomez et al., 2014, Walakira et al., 2017); iii) the differences in the biology and pathogenesis of *S. mansoni* and *S. haematobium* (Adedokun et al., 2018; Hanton et al., 2022); iv) the study design and the sample size (Mewamba et al., 2021a).

Our results showing significantly higher concentration of IL-13 in children with high *S. mansoni* burden compared to other are in agreement with those of Long et al. (2015) who reported higher concentration of IL-13 in people infected with *S. japonicum* and having liver fibrosis compared to those with normal liver. Other studies highlighted the influence of high concentration of IL-13 during the progression of schistosomiasis due to *S. mansoni* (Mutengo et al., 2018). These results highlighted an up-regulation of IL-13 during schistosome infections. These molecules may probably play a role during schistosome infections by eliminating schistosome eggs lodged in mammals' tissue (Gazzinelli and Colley, 1992).

The significantly higher concentration of IgE in children with high *S. mansoni* burden compared to those with light or no schistosome infections are in line with results of Figueiredo et al. (2012). These results suggest that the concentration of total IgE may have an impact on the intensities of *S. mansoni* infections. This hypothesis is strengthened by the strong, positive and significant correlation obtained between the concentration of IgE and the amount of CCA released in urine. However, results of the present study do not agree with those of previous authors who associated high concentration of IgE with the capacity of children to be resistant to schistosome infections (Hagan et al., 1991; Rihet et al., 1991; Demeure et al., 1993; Ndombi et al., 2018). The discrepancies between results of these studies could be related to the lack of discrimination between schistosome specific IgE and other allergen IgE (Grant et al., 2012). Moreover, higher concentration of IgE in children bearing high infection intensities could result from an important immune response against large numbers of *S. mansoni* eggs. Indeed, when *Schistosoma* eggs are released in mammalian hosts, the immune response of type Th2 induces an increasing plasma concentration of IgE.

Although no multiple correction test was performed during our association studies, results of QTDT showing an association between the allele A of SNP rs2069739 of *IL13* gene with an increased risk of having low plasma concentrations of IL-13 are consistent with those of Choto et al. (2021) who linked some polymorphisms of *IL13* gene with schistosome infections. These results suggest that *IL13* variants may probably affect the production or the plasma concentration of IL-13. Moreover, the association between the G allele of SNP rs2243283 of *IL4* gene with an increased risk of having low concentration of IL-10 indicate that this polymorphism may influence the production IL-10. This hypothesis is strengthened by results of Mitchell et al. (2017). Indeed, after knockout of the alpha receptor of *IL4* genes, these authors observed an inhibition of IL-10 production; indicating that polymorphism within *IL4* gene may play key role during IL-10 production. During schistosome infections, the penetration and migration of schistosomules and the deposit of eggs in tissues induce immune responses of Th1 and Th2 (Grzych et al., 1991; Perrigoue et al., 2008; Egesa et al., 2018). In this process, the activation of different pathways leads to the production of not only pro-inflammatory cytokines like IFN-γ in Th1 pathway, but also inflammatory cytokines like IL-4, IL-10 and IL-13 in Th2 pathway. For instance, IL-13 is involved not only in the regulation of Th2 immune response, but also in the production of STAT6 which in turn play important role in the switching of immunoglobin classes (Goenka and Kaplan, 2011). Moreover, Th2 immune response is essential in the development of a protective immunity against schistosome infections. This observation was supported by the fact that the resistance to schistosome infections is characterized by strong Th2-mediated immunity (Milner et al., 2010). Nevertheless, as Th2 immune response is associated with the development of immunopathology including inflammation, granuloma and fibrosis formation (Fairfax et al., 2012; Bourke et al., 2013), the Th1 pathway has been reported to limit the observed immunopathological developments (Arnaud et al., 2008). In addition to that, IL-10 down-regulates immune responses in long standing schistosome infections (Joseph et al., 2004).

The absence of association between polymorphism at *IL13* and IgE level is in agreement with results of Grant et al. (2012). These results could be explained by the fact that the IgE measured in the present study result from a combination of IgE specific to *S. mansoni* infections and that produced in response to other helminth infections or antigens. This hypothesis is strengthened by the fact that the study sites are endemic to schistosomiasis and soil transmitted helminthes (Tchuem Tchuenté et al., 2012; Kamdem et al., 2022). These soil transmitted helminths can also induced the production of IgE. Our results showing no association between polymorphisms within *IL10* gene and IgE do not agree with those of Grant et al. (2011). The discrepancies between results of these studies could be related to the differences in the phenotypes used. In the present study, children were phenotyped using the CCA while egg count was used in previous ones.

The positive and significant correlations recorded between the amount of CCA with the concentrations of either IL-13 or IFN-γ or IgE indicate that the plasma concentration of IgE as well as those of the two cytokines could be related to the infection intensities inferred from the amount of CCA.

## 5 Conclusion

This study revealed that the C allele of SNP rs3024974 of *STAT6* gene was associated with an increased risk of bearing high *S. mansoni* burden while the C allele of SNP rs1800871 of *IL10* gene decrease the risk of bearing such phenotype. It showed that the alleles C of SNP rs3024974 of *STAT6* gene and C of SNP rs1800871 of *IL10* gene may respectively increase and decrease the risk of bearing high burden of *S. mansoni* within the Cameroonian populations. The alleles A of SNP rs2069739 of *IL13* gene and G of SNP rs2243283G of *IL4* gene were significantly associated respectively with low concentrations of IL-13 and IL-10. Results of this study showed that host genetic polymorphisms have some impacts not only in the outcome of (high or low worm burden) *S. mansoni* infections, but also on the plasma concentrations of some cytokines.

The following are the supplementary data related to this article.

Supplementary Table S1 Power of the study according to genetic markers. Supplementary Table S1

Supplementary Table S2 Frequency of gene polymorphisms within populations of Makenene and Nom-Kandi. Supplementary Table S2

Supplementary Table S3 Results of TDT associating polymorphisms within *IL4, IL10, IL13, IFNG* and *STAT6* genes and infection intensities in *S. mansoni* infected children from Makenene and Nom-Kandi. Supplementary Table S3

## CRediT authorship contribution statement

**Estelle Mezajou Mewamba:** Conceptualization, Funding acquisition, Data curation, Formal analysis, Methodology, Writing – original draft. **Harry Noyes:** Conceptualization, Data curation, Methodology, Writing – review & editing. **Arnol Auvaker Zebaze Tiofack:** Data curation, Formal analysis, Methodology, Writing – original draft. **Rolin Mitterran Ndefo Kamga:** Data curation, Formal analysis, Methodology. **Cyrille Nguemnang Kamdem:** Data curation, Formal analysis, Methodology. **Loic Edmond Tekeu Mengoue:** Data curation, Formal analysis, Methodology. **Elvis Ofon:** Data curation, Formal analysis, Software. **Romuald Isaka Kamwa Ngassam:** Data curation, Methodology, Supervision. **Oscar Nyangiri:** Data curation, Formal analysis, Methodology, Software. **Bruno Bucheton:** Funding acquisition, Conceptualization, Writing - review & editing. **Flobert Njiokou:** Conceptualization, Supervision, Writing – review & editing. **Macaire Hilaire Womeni:** Supervision, Writing – review & editing. **Enock Matovu:** Conceptualization, Funding acquisition, Writing – review & editing. **Annette MacLeod:** Conceptualization, Funding acquisition, Writing – review & editing. **Gustave Simo:** Conceptualization, Funding acquisition, Supervision, Writing – original draft, Writing – review & editing.

## Declaration of Competing Interest

The authors declare that they have no conflict of interest.

## Data Availability

All data generated and/or analyzed are included in this article and in supplemetary tables.
