# Supplementary material for: Association between polymorphisms of IL4, IL13, IL10, STAT6 and IFNG genes, cytokines and immunoglobulin E levels with high burden of Schistosoma mansoni in children from schistosomiasis endemic areas of Cameroon
Source: Infect Genet Evol. 2023 Jul;111:105416. doi: 10.1016/j.meegid.2023.105416 (PMC10167540; doi:10.1016/j.meegid.2023.105416)
Supplement: Supplementary Table S1 — Power of the study according to genetic markers. [file mmc1.docx]

**Table S1: Power of the study according to genetic markers**

| **Gene** | **Marker** | **Minor allele frequency** | **Power of the study** |
| --- | --- | --- | --- |
| *IL13* | rs2069743 | 0.23 | 87.9% |
| *IL4* | rs2243250 | 0.26 | 87.5% |
| *IFNG* | rs2430561 | 0.19 | 88.3% |
| *IL13* | rs1800925 | 0.43 | 85.9% |
| *IL13* | rs7719175 | 0.18 | 88.4% |
| *IL13* | rs20541 | 0.12 | 89% |
| *IL13* | rs2069739 | 0.40 | 86.2% |
| *IL13* | rs1295687 | 0.33 | 86.8 |
| *IL10* | rs1800871 | 0.45 | 85.7% |
| *IL10* | rs1800872 | 0.46 | 85.6% |
| *IL10* | rs1800896 | 0.34 | 86.7% |
| *IL4* | rs2243268 | 0.34 | 86.7% |
| *IL4* | rs2243283 | 0.24 | 87.7% |
