# Supplementary material for: Association between polymorphisms of IL4, IL13, IL10, STAT6 and IFNG genes, cytokines and immunoglobulin E levels with high burden of Schistosoma mansoni in children from schistosomiasis endemic areas of Cameroon
Source: Infect Genet Evol. 2023 Jul;111:105416. doi: 10.1016/j.meegid.2023.105416 (PMC10167540; doi:10.1016/j.meegid.2023.105416)
Supplement: Supplementary Table S2 — Frequency of gene polymorphisms within populations of Makenene and Nom-Kandi. [file mmc2.docx]

**Table S2:** Frequency of gene polymorphisms within populations of Makenene and Nom-Kandi.

| **Polymorphisms** | **Genotypes** | **Frequencies on parents** | **P-value of Hardy Weinberg equilibrium test** |
| --- | --- | --- | --- |
| **rs3024974** | T/T | 1 |  |
|  | T/C | 34 | 0.69 |
|  | C/C | 135 |  |
| **rs2069743** | G/G | 13 |  |
|  | G/A | 52 | 0.08 |
|  | A/A | 105 |  |
| **rs2243250** | C/C | 15 |  |
|  | C/T | 57 | 0.15 |
|  | T/T | 98 |  |
| **rs2430561** | T/T | 6 |  |
|  | T/A | 54 | 1 |
|  | A/A | 110 |  |
| **rs1800925** | T/T | 29 |  |
|  | T/C | 89 | 0.44 |
|  | C/C | 52 |  |
| **rs7719175** | G/G | 9 |  |
|  | G/T | 42 | 0.06 |
|  | T/T | 119 |  |
| **rs20541** | A/A | 4 |  |
|  | A/G | 36 | 0.49 |
|  | G/G | 130 |  |
| **rs2069739** | G/G | 30 |  |
|  | G/A | 75 | 0.34 |
|  | A/A | 65 |  |
| **rs1295687** | C/C | 21 |  |
|  | C/G | 71 | 0.49 |
|  | G/G | 78 |  |
| **rs1800871** | C/C | 31 |  |
|  | T/C | 94 | 0.16 |
|  | T/T | 35 |  |
| **rs1800872** | A/A | 39 |  |
|  | A/C | 78 | 0.35 |
|  | C/C | 53 |  |
| **rs1800896** | G/G | 21 |  |
|  | G/A | 75 | 0.74 |
|  | A/A | 74 |  |
| **rs2243268** | C/C | 18 |  |
|  | C/A | 80 | 0.61 |
|  | A/A | 72 |  |
| **rs2243283** | C/C | 11 |  |
|  | C/G | 60 | 0.68 |
|  | G/G | 99 |  |
