# Supplementary material for: Association between polymorphisms of IL4, IL13, IL10, STAT6 and IFNG genes, cytokines and immunoglobulin E levels with high burden of Schistosoma mansoni in children from schistosomiasis endemic areas of Cameroon
Source: Infect Genet Evol. 2023 Jul;111:105416. doi: 10.1016/j.meegid.2023.105416 (PMC10167540; doi:10.1016/j.meegid.2023.105416)
Supplement: Supplementary Table S3 — Results of TDT associating polymorphisms within IL4, IL10, IL13, IFNG and STAT6 genes and infection intensities in S. mansoni infected children from Makenene and Nom-Kandi. [file mmc3.docx]

**Table S3:** Results of TDT associating polymorphisms within *IL4, IL10, IL13, IFNG* and *STAT6* genes and infection intensities in *S. mansoni* infected children from Makenene and Nom-Kandi

| ***Population of Makenene*** | | | | | | **Additive model** | | | **Recessive model** |  |  |
| --- | --- | --- | --- | --- | --- | --- | --- | --- | --- | --- | --- |
| **Gene** | **Marker** | **Risk Allele** | **Allele frequency ^a^** | **A1** | **MAF** | **Informative families ^b^** | **Z Score ^c^** | **P** | **Informative families ^b^** | **Z Score ^c^** | **P** |
| ***STAT6*** | rs3024974 | **C**** | **0.9** | **T** | **0.1** | **29** | **2.80** | **0.004** | **27** | **2.59** | **0.009** |
| *IL13* | rs2069743 | A | 0.79 | G | 0.21 | 43 | 1.73 | 0.08 | 38 | 1.05 | 0.45 |
| *IL4* | rs2243250 | T | 0.76 | C | 0.24 | 43 | 0.56 | 0.57 | 35 | -0.65 | 0.51 |
| *IFNG* | rs2430561 | A | 0.83 | T | 0.17 | 37 | 1.18 | 0.24 | 34 | 0.692 | 0.48 |
| *IL13* | rs1800925 | C | 0.60 | T | 0.40 | 60 | 0.57 | 0.57 | 47 | -0.39 | 0.69 |
| *IL13* | rs7719175 | T | 0.84 | G | 0.16 | 35 | 0.79 | 0.43 | 33 | 1.07 | 0.28 |
| *IL13* | rs20541 | G | 0.88 | A | 0.12 | 33 | -0.63 | 0.53 | 32 | -0.66 | 0.51 |
| *IL13* | rs2069739 | A | 0.60 | G | 0.4 | 66 | -0.58 | 0.56 | 48 | -1.49 | 0.14 |
| *IL13* | rs1295687 | G | 0.68 | C | 0.32 | 55 | -0.17 | 0.86 | 44 | -0.29 | 0.77 |
| ***IL10*** | **rs1800871** | **T** | **0.53** | **C*** | **0.47** | 50 | 1.57 | 0.16 | **26** | **-2.78** | **0.005** |
| *IL10* | rs1800872 | C | 0.53 | A | 0.47 | 58 | -1.9 | 0.06 | 41 | -1.69 | 0.09 |
| *IL10* | rs1800896 | A | 0.65 | G | 0.35 | 52 | 1.9 | 0.05 | 39 | 1.56 | 0.12 |
| *IL4* | rs2243268 | A | 0.64 | C | 0.36 | 65 | -0.52 | 0.60 | 55 | -0.94 | 0.34 |
| *IL4* | rs2243283 | G | 0.74 | C | 0.26 | 50 | -0.66 | 0.51 | 43 | -1.42 | 0.17 |
| ***Population of Nom-Kandi*** | | | | | |  |  |  |  |  |  |
| *STAT6* | rs3024974 | C | - | - | - | - | - | - | - | - | - |
| *IL13* | rs2069743 | A | 0.72 | G | 0.28 | 12 | 0.40 | 0.68 | 11 | 0.19 | 0.84 |
| *IL4* | rs2243250 | T | 0.69 | C | 0.31 | 14 | -0.97 | 0.33 | 12 | -1.02 | 0.30 |
| *IFNG* | rs2430561 | A | 0.76 | T | 0.24 | 11 | -0.04 | 0.97 | 10 | 0.08 | 0.93 |
| *IL13* | rs1800925 | T | 0.53 | T | 0.53 | 15 | 1.28 | 0.20 | 11 | 0.49 | 0.62 |
| *IL13* | rs7719175 | T | 0.74 | G | 0.26 | 12 | -0.40 | 0.68 | 10 | -0.53 | 0.6 |
| *IL13* | rs20541 | G | - | - | - | - | - | - | - | - | - |
| *IL13* | rs2069739 | A | 0.59 | G | 0.41 | 10 | -1.04 | 0.29 | - | - | - |
| *IL13* | rs1295687 | G | 0.57 | C | 0.43 | 10 | 1.58 | 0.11 | - | - | - |
| *IL10* | rs1800871 | C | 0.64 | C | 0.36 | 17 | -0.05 | 0.95 | 16 | -1.44 | 0.15 |
| *IL10* | rs1800872 | C | 0.54 | A | 0.46 | 15 | 0.52 | 0.60 | 13 | -0.12 | 0.90 |
| *IL4* | rs2243268 | A | - | - | - | - | - | - | - | - | - |
| *IL10* | rs1800896 | A | 0.72 | G | 0.28 | 11 | -1.04 | 0.29 | 10 | -0.96 | 0.34 |
| *IL4* | rs2243283 | G | 0.84 | C | 0.16 | 11 | -1.15 | 0.25 | 11 | -1.14 | 0.25 |

a: Calculated from the genotype of parents.**;** b: Families with at least one heterozygous parent; c: Z =(S - E (S))/Var(S), where S is the test statistic (i.e. observed), E(S) is the expected value according to the null hypothesis (H0), and Var(S) is the variance of the statistic test according to H0; A1: Minor allele code, MAF: Minor allele frequency; **: allele associated with an increase of bearing high worm burden, *: allele associated with a decrease of bearing high worm burden.
